# AI and Chatbot-Supported Interventions for Physical Activity and Obesity-Related Lifestyle Behaviors: Scoping Review With Attention to Family Involvement

**DOI:** 10.2196/98889

**Published:** 2026-07-24

**Authors:** Qianxia Jiang, Xiayu Summer Chen, Dev Patel, Keith Brazendale, Sualba Alejandro

**Affiliations:** 1 Department of Health Sciences, University of Central Florida, 4364 Scorpius St, Orlando, FL, 32816, United States, 1 8609318880; 2 School of Social Work, University of Central Florida, Florida, United States; 3 College of Medicine, University of Central Florida, Florida, United States

**Keywords:** artificial intelligence, chatbot, family-based intervention, physical activity, pediatric obesity, lifestyle behaviors

## Abstract

**Background:**

AI-enabled chatbots and related conversational systems can facilitate human–computer interaction through natural language, personalization, and automated support. In pediatric health promotion, these tools have the potential to provide scalable and flexible approaches to support physical activity (PA) and related lifestyle behavior change within family contexts. However, evidence regarding AI and chatbot-supported interventions for PA and obesity-related lifestyle behaviors among children and adolescents remains limited, and the extent to which these interventions involve parents, caregivers, or families has not been clearly characterized.

**Objective:**

This scoping review aimed to provide an up-to-date overview of how AI and chatbot-supported interventions are designed, delivered, and evaluated for PA and obesity-related lifestyle behaviors among children and adolescents, with attention to technology characteristics, family involvement, delivery platforms, outcomes, and research gaps.

**Methods:**

In accordance with PRISMA-ScR (Preferred Reporting Items for Systematic Reviews and Meta-Analyses extension for Scoping Reviews) guideline, 7 databases (PubMed, Web of Science, APA PsycINFO, Academic Search Complete, CINAHL Ultimate, IEEE Xplore, and Scopus) were searched through February 2026. Two reviewers independently conducted title and abstract screening, followed by full-text screening in Rayyan (Qatar Computing Research Institute). Eligible studies involved children, adolescents, or families; evaluated an AI-enabled chatbot, conversational agent, or related digital system; and addressed PA, exercise, sedentary behavior, screen time, obesity, overweight, or weight management. Diet, sleep, and other lifestyle outcomes were extracted when reported within otherwise eligible studies. Data were extracted and synthesized narratively in accordance with research objectives.

**Results:**

Of 2730 records identified, 14 studies met inclusion criteria. Most were published in 2023 or later (n=12, 85.7%) and spanned 10 countries. Mobile app delivery was most common (n=9, 64.3%). AI approaches included rule-based chatbots, hybrid personalization systems, generative AI, and single studies using recommender systems or computer vision. A total of 10 studies (71.4%) included a specific parent, caregiver, or family component. Common intervention features were personalized feedback (n=10, 71.4%), self-monitoring (n=9, 64.3%), and education (n=9, 64.3%). PA was the most common target behavior, often within broader obesity or healthy lifestyle interventions. Among 8 studies reporting direct PA or fitness outcomes, 4 showed significant improvement, 2 found no significant change, and 2 reported mixed or indirect findings. Feasibility, acceptability, usability, and engagement findings were generally favorable across studies, but cultural tailoring was reported in only one study.

**Conclusions:**

AI and chatbot-supported interventions for pediatric PA and obesity-related lifestyle behaviors represent a rapidly emerging but still early-stage field. Family involvement varies considerably across interventions and should be more clearly conceptualized and evaluated in future studies.

## Introduction

Childhood physical inactivity (PA) and obesity are persistent, high-impact challenges across the life course and often cluster within families through shared environments, routines, and resources [1]. World Health Organization (WHO) guidelines recommend that children and adolescents accumulate an average of at least 60 minutes per day of moderate-to-vigorous PA, while also reducing sedentary time [2]. Yet recent global surveillance indicates that PA levels remain low across childhood and adolescence. Global Matrix 4.0 data from 57 countries suggest that only about 27%-33% of children and adolescents aged 5-17 years meet recommended activity levels [3], and the WHO reports that 81% of adolescents aged 11-17 years are insufficiently active worldwide [4].

Pediatric overweight and obesity also represent a growing global public health challenge. In 2022, more than 390 million children and adolescents aged 5-19 years worldwide were living with overweight, including more than 160 million living with obesity. The prevalence of overweight, including obesity, among children and adolescents aged 5‐19 years increased from 8% in 1990 to 20% in 2022, while the prevalence of obesity alone increased from 2% to 8% [5]. Important regional differences are also evident. Latin America and the Caribbean, the Middle East and North Africa, and North America have among the highest prevalence of overweight, while the largest numbers of affected children and adolescents are concentrated in East Asia and the Pacific, Latin America and the Caribbean, and South Asia [6]. These patterns reflect broader behavioral, environmental, and structural determinants of health and reinforce the need for accessible and scalable approaches to support healthy behaviors among children, adolescents, and families [1].

Families play a central role in shaping children’s health behaviors [7]. Caregivers influence opportunities for PA through role modeling, encouragement, co-participation, transportation, and the organization of routines [8]. Therefore, family-based approaches are widely recognized as an important strategy for supporting healthy lifestyle behaviors and pediatric obesity prevention and treatment [7]. At the same time, participation in intensive family-based programs can be difficult to sustain because of barriers such as cost, scheduling demands, transportation, workforce limitations, and unequal access to care [7]. These challenges are particularly relevant for underserved families and have increased interest in more flexible and scalable approaches to family-centered behavior change support.

Digital health and mHealth have rapidly expanded as a delivery channel for behavior change interventions, supported by widespread mobile device access and increasing interest from health systems in scalable, low-cost approaches [9,10]. Within this broader digital health landscape, conversational tools such as chatbots and virtual agents represent one emerging delivery format that may support health promotion through interactive communication, tailored prompts, and ongoing engagement [9,10]. However, for pediatric PA and relevant lifestyle behaviors, the literature remains scattered across diverse populations, platforms, intervention designs, and outcomes.

AI has further expanded the potential of digital health interventions for children and families by enabling more tailored, interactive, and responsive (ie, real-time) support [11,12]. In contrast to static digital content, AI-enabled systems such as chatbots and conversational agents may help deliver intervention components based on user input, preferences, or real-time data [13]. In pediatric lifestyle interventions, these features may be especially relevant because children’s PA and related behaviors are shaped by changing routines, developmental needs, and family context [12]. However, the evidence base remains fragmented across platforms, target behaviors, and outcome measurement strategies [14].

Despite growing interest in AI-enabled digital health tools, few studies have synthesized the current evidence on AI and chatbot-supported interventions for pediatric PA and obesity-related lifestyle behaviors while examining the extent and nature of family involvement [10,12,13]. Previous reviews have examined conversational agents in health care broadly, chatbot-supported lifestyle interventions across age groups, or the feasibility and acceptability of chatbots for nutrition and PA promotion among adolescents [10,12,13]. However, these reviews often aggregate adult and pediatric populations, emphasize individual-level interventions, or examine digital health more broadly without distinguishing AI-driven systems from traditional mobile apps [10,12]. Moreover, family engagement within digital interventions is inconsistently conceptualized and operationalized, ranging from passive caregiver monitoring to active, dyadic, or multigenerational participation [12]. Questions also remain regarding cultural responsiveness, equity considerations, accessibility for underserved families, and safeguards related to data privacy, algorithmic bias, transparency, and accountability [12]. To address these gaps, the present review focuses specifically on AI and chatbot-supported interventions for pediatric PA and related lifestyle behaviors, with particular attention to family engagement, technology characteristics, cultural tailoring, and implementation gaps.

Given the rapid evolution of AI technologies and the heterogeneity of study designs, including pilot feasibility trials, randomized controlled trials, and early-stage implementation studies, a scoping review is warranted. Thus, the objective of this scoping review was to map and characterize AI and chatbot-supported interventions targeting pediatric PA and obesity-related lifestyle behaviors. Priority areas of focus are (1) technology type and AI features, (2) delivery platforms and intervention components, (3) definition and operationalization of family engagement, (4) cultural tailoring and bilingual delivery strategies, and (5) the characteristics of the existing evidence base, including study designs, outcomes, and gaps to inform future research and equitable implementation.

## Methods

### Study Design

A formal review protocol was not prospectively registered. Before screening began, the research team developed the review objectives, eligibility criteria, search strategy, screening procedures, and standardized data-extraction form. The complete database search strategies are provided in Multimedia Appendix 1, and the completed PRISMA-ScR (Preferred Reporting Items for Systematic Reviews and Meta-Analyses extension for Scoping Reviews) checklist is provided in Checklist 1 [15]. The review aimed to map and synthesize the existing literature on AI and chatbot-supported interventions targeting pediatric PA and obesity-related lifestyle behaviors, with particular attention to the extent and nature of family involvement.

### Search Strategy

#### Information Sources

A comprehensive literature search was conducted across 7 electronic databases, including PubMed, Web of Science, APA PsycINFO, Academic Search Complete, CINAHL Ultimate, IEEE Xplore, and Scopus. The final search was performed on February 8, 2026. Searches were limited to English-language peer-reviewed publications published within the past 10 years. This timeframe was selected to capture contemporary AI-enabled and chatbot-supported technologies relevant to current practice, given the rapid evolution of conversational-agent platforms, natural language processing, adaptive personalization, wearable integration, and generative AI. Earlier digital interventions may be less comparable with the systems currently used in pediatric health promotion. The search was limited to peer-reviewed publications indexed in the selected databases. A formal gray-literature search and supplementary backward or forward citation tracking were not conducted.

#### Search Terms

Search strategies were developed to capture three primary domains: (1) AI-enabled conversational technologies, (2) PA and obesity-related outcomes, and (3) child and family populations. Terms for AI technologies included chatbot*, chat bot*, conversational agent*, virtual agent*, relational agent*, virtual assistant*, AI coach*, ChatGPT (OpenAI), large language model*, LLM*, and generative AI. Health-related terms included physical activity*, exercise*, obese*, obesity, overweight, weight management, obesity prevention, sedentary, and screen. Population terms included child*, adolescent*, youth, teen*, pediatric*, family*, parent*, caregiver*, family-based, and parent-based. Database-specific syntax adjustments were applied as needed, including title and abstract field restrictions and wildcard limits. The detailed search history was provided in supplementary material (Multimedia Appendix 1).

Review articles, systematic reviews, scoping reviews, and meta-analyses were excluded during the search process where possible using database filters.

### Eligibility Criteria

Eligibility criteria were guided by the population, intervention, comparator, outcomes, and study design (PICOS) framework. Studies were included if they: (P) involved children or adolescents aged 0‐18 years or parents, caregivers, or families supporting the health behaviors of a child or adolescent within this age range; (I) evaluated an AI-enabled chatbot, conversational agent, or related digital system designed to support PA or obesity-related lifestyle behaviors; (C) included any comparator, such as usual care or an alternative intervention, or no comparator; (O) reported outcomes related to PA or exercise, sedentary behavior or screen time, obesity, overweight, or weight management; and (S) reported empirical quantitative, qualitative, or mixed methods findings.

Studies were eligible if they involved children or adolescents or parents, caregivers, or families supporting the health behaviors of children or adolescents. Direct parent or caregiver involvement was not required because family involvement was examined as an analytic dimension rather than an eligibility criterion.

Studies were excluded if they described general digital health tools without an automated conversational, chatbot, AI-enabled, adaptive, or personalized support component, were purely technical development papers without behavioral outcomes, or were nonempirical publications such as editorials, commentaries, protocols, or review articles. Only studies published in English were included.

### Study Selection

All retrieved records were imported into Rayyan (Qatar Computing Research Institute), a web-based systematic review management platform [16]. Rayyan was used to manage records, identify potential duplicates, and support blinded title and abstract screening. Potential duplicates identified through bibliographic metadata were manually reviewed and confirmed before removal. Final eligibility decisions were made by the reviewers rather than by the software. Title and abstract screening were conducted independently by 2 reviewers (DP and SA), who were blinded to each other’s decisions during the initial screening phase. At this stage, records were excluded if they were clearly unrelated to pediatric, adolescent, or family populations; did not involve AI-enabled chatbots, conversational agents, or related automated digital systems; did not address PA, obesity, sedentary behavior, weight management, or related lifestyle behaviors; or were clearly nonempirical publications. Records were retained for full-text review when eligibility could not be determined from the title and abstract alone. Full-text screening was then conducted independently by 2 reviewers (DP and SA) using the complete eligibility criteria. At this stage, specific reasons for exclusion were recorded. Articles were excluded during full-text review if they did not report PA or related lifestyle outcomes, did not include children, adolescents, parents, caregivers, or families, did not include an AI-enabled or chatbot-supported intervention component, or provided insufficient information to determine eligibility. Disagreements were resolved through discussion, and unresolved disagreements were adjudicated by a third reviewer (QJ). Interrater reliability was assessed using Cohen kappa (κ=0.81).

### Data Extraction and Synthesis

Data were extracted using a standardized form developed for this review. Extracted variables included publication and study characteristics (author, year, country, design, and sample size); participant characteristics (target population, age, sex, and family involvement); intervention characteristics (intervention type, delivery platform, duration, comparator, wearable or sensor use, and behavior change components); AI-related features (AI or automation role, intervention system or platform, reported AI technique or automation logic, input data type, and AI tasks or outputs); theoretical foundations; and reported outcomes, including feasibility, acceptability, usability, engagement, PA, and related lifestyle outcomes, secondary outcomes, and key findings. Extracted data were synthesized narratively to characterize the scope of the evidence and identify patterns across study design, intervention delivery, AI functionality, family engagement, and outcomes.

### Critical Appraisal

A formal critical appraisal or methodological quality assessment was not conducted. The purpose of this scoping review was to map the characteristics, applications, and gaps within an emerging and heterogeneous evidence base rather than to assess the certainty of evidence or estimate pooled intervention effects.

## Results

### Overview

**Figure 1.**
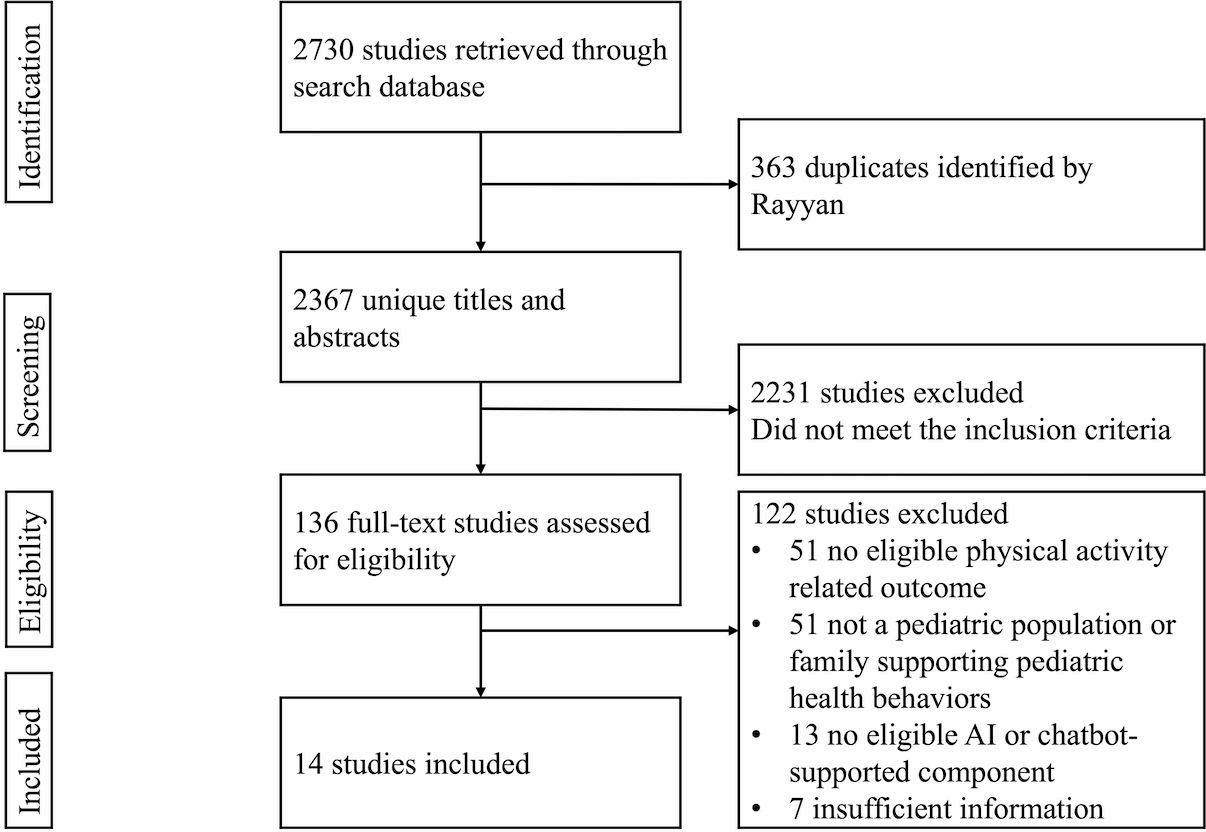
PRISMA (Preferred Reporting Items for Systematic Reviews and Meta-Analyses) flowchart for study selection process.

Figure 1 presents the PRISMA (Preferred Reporting Items for Systematic Reviews and Meta-Analyses) flow diagram. The initial database search identified 2730 records. After removing 363 duplicate records, 2367 unique articles remained for title and abstract screening. Of these, 2231 were excluded because they were clearly outside the review scope. A total of 136 full-text articles were assessed for eligibility. During full-text review, 122 articles were excluded for the following primary reasons: no PA or related lifestyle outcome, not child-, adolescent-, parent-, caregiver-, or family-focused, no AI-enabled or chatbot-supported component, or insufficient information to determine eligibility. Ultimately, 14 studies met the inclusion criteria and were included in the final sample (Figure 1).

### Study Overview

A total of 14 original studies were included in this scoping review [17-30] (Table 1). The studies were published between 2021 and 2026, with 12 of the 14 published in 2023 or later. The 14 studies were conducted across 10 countries. The United States and South Korea were the most frequently represented settings (n=3 each), followed by one study each from Turkey, Spain, Italy, the United Kingdom, Belgium, Switzerland, Greece, and Canada. Although the included studies were geographically diverse, all were conducted in middle- or high-income contexts.

**Table 1.** Characteristics of included studies.

Study and year	Country	Study design	Sample size
Aydemir (2025) [17]	Turkey	Pre-post feasibility study with intervention and control groups	26 parent-child dyads (13 intervention and 13 control)
de Arriba Muñoz et al (2026) [18]	Spain	Prospective single-arm observational pre-post study	40 families or pediatric patients
Emerson et al (2026) [30]	United States	Three-phase qualitative adaptation study	22 total (phase 1: n=6; phase 2: n=4; phase 3: n=12)
Heerman et al (2024) [19]	United States	Multicenter randomized clinical trial	900 parent-infant pairs
Larizza et al (2023) [22]	Italy	App development+pilot usability or acceptability study	13 children
Moore et al (2024) [23]	United Kingdom	Co-design study with mixed methods evaluation	9 co-design participants; 46 evaluation participants
Oh et al (2022) [24]	South Korea	Randomized controlled trial	24 adolescents
Peuters et al (2024) [25]	Belgium	Quasi-randomized controlled trial+process evaluation interviews	279 adolescents; interviews n=13
Stasinaki et al (2021) [26]	Switzerland	Randomized controlled trial	41 recruited; analysis n=31 at baseline after exclusions or dropout
Tan et al (2026) [27]	United States	Single-arm pilot feasibility study	172
Zarkogianni et al (2023) [29]	Greece	Feasibility pilot trial	50 children
Kim et al (2025) [20]	South Korea	User-centered design or development and usability study	Needs survey n=96; interviews n=30; UI usability n=76
Kang et al (2026) [21]	South Korea	Descriptive comparative cross-sectional survey study	160 total (80 childhood or adolescent cancer survivors >80 parents)
Willms and Liu (2024) [28]	Canada	Autoethnographic feasibility case study or development paper	No human participants; content development case study

Among included studies, 3 studies were randomized controlled trials [19,24,26], one was quasi-randomized [25], one was a pre-post feasibility study with a comparison group [17], and 3 were single-arm or uncontrolled pilot or pre-post studies [18,22,27]. The remaining 6 were noneffectiveness-focused designs, including one qualitative adaptation study [30], one co-design mixed methods study [23], 2 development or usability studies [20,28], one cross-sectional survey [21], and one autoethnographic feasibility or development paper [29]. Sample sizes ranged from 9 to 900 participants, with most studies enrolling relatively small samples; only one large, randomized trial included more than 500 participants [19].

### Participant and Family Characteristics

The 14 studies targeted a broad range of pediatric populations, including infants, school-aged children, and adolescents (Table 2). Most studies focused on school-aged children or adolescents, whereas one large trial focused on infants from birth to 24 months [19]. Several studies targeted specific clinical or high-need populations, including youth with autism spectrum disorder (n=2) [17,30], children or adolescents with obesity or overweight (n=5) [18,22,24,26,29], childhood or adolescent cancer survivors (n=1) [21], and adolescents facing barriers to PA or living in under-resourced settings (n=2) [23,27].

**Table 2.** Participant and family characteristics of included studies.

Study and Year		Target population or unit of Intervention	Population characteristics or special group	Child age	Parent age	Sex (female), n (%)	Language	Cultural tailoring
Aydemir (2025) [17]		Parent-child dyads	Children with autism spectrum disorder (ASD)	Intervention: mean 14.38 (SD 3.06) Control: mean 13.92 (SD 3.52)	Intervention: mean 42.84 (SD 7.70) Control: mean 44.07 (SD 7.95)	Children: intervention 6/13 (46%), control 8/13 (62%) Parents: intervention 5/13 (38%), control 4/13 (31%)	Likely Turkish / ChatGPT (OpenAI)-supported text prompts	NR^a^
de Arriba Muñoz et al (2026) [18]		Family (caregiver+ child or adolescent)	Children or adolescents with obesity receiving GLP-1^b^ therapy	Mean 14.05 (SD 1.04) years	NR	25/40 (62.5%)	Spanish	NR
Emerson et al (2026) [30]		Youth with ASD and caregivers; providers or therapists	Youth with autism spectrum disorder at risk of obesity	Youth with ASD; exact ages not reported in abstract	NR	NR	English	NR
Heerman et al (2024) [19]		Parent-child dyads	Infants from racially and ethnically diverse US families	Birth-24 months follow-up	Adults ≥18 years	Children: intervention: 243/449 (54.1%) Control: 233/451 (51.7%) female	English and Spanish	Yes; all content translated into Spanish and assessed for cultural appropriateness
Larizza et al (2023) [22]		Child and parent	Children with obesity	Age: 6‐12 years Mean 9.3 (SD 1.3) years	NR	5/13 (38.5%)	Italian context	NR
Moore et al (2024) [23]		Adolescents	Adolescents facing barriers to physical activity	11‐13 years (co-design) Adolescents in evaluation	NR	Co-design group 5/9 (56%)	English	Personalization emphasized; no cultural tailoring reported
Oh et al (2022) [24]		Adolescents	Adolescents with obesity	10‐17 years Mean 13.2 (SD 3.6)	NR	4/24 (16.7%)	Korean context	NR
Peuters et al (2024) [25]		Adolescents	General population adolescents aged 12‐15 during COVID-19 restrictions	12‐15 years	NR	NR	Dutch	Human-centered development; no explicit cultural tailoring
Stasinaki et al (2021) [26]		Youth or adolescents	Youth with overweight or obesity receiving specialist obesity care	10‐18 years Median baseline age 13.6 years (range 10.9 - 16.9)	NR	13/31 (41.9%)	German/Swiss context	NR
Tan et al (2026) [27]		Children or adolescents	Primarily sixth-grade students in socioeconomically disadvantaged schools	Mostly 12 years Range 11‐15 years	NR	96/172 (55.8%)	English	NR
Zarkogianni et al (2023) [29]		Child and parent	Overweight or obese children	Mean 10.5 (SD 2.00) years	NR	26/50 (52%)	Greek context	NR
Kim et al (2025) [20]		Adolescents + parents + school health teachers	Middle school adolescents in school-based health management context	First-year middle school students or adolescents	Range 30‐59 years	Students 23/36 (65%) Parents 25/40 (83%) Teachers 30/30 (100%)	Korean	Designed for Korean school context
Kang et al (2026) [21]		Childhood or adolescent cancer survivors and parents	Childhood or adolescent cancer survivors (aged 10‐16 years) recruited via the Korean Pediatric Cancer Foundation; survivor-parent perspectives	Mean 12.1 (SD 2.0) years Eligibility 10‐16 years	Mean 44.7 (SD 4.2) years	CACSs^c^ 41/80 (51.2%); Parent respondents 76/80 (95.0%)	Korean	South Korean childhood cancer survivorship context; no explicit cultural tailoring intervention because no chatbot program was deployed
Willms and Liu (2024) [28]		Parents supporting child physical activity (intended target)	Family-based PA^d^ content development for children aged 8‐12 and parents	Intended target: children 8‐12 years	NR	NR	English	NR

a NR: not reported.

b GLP-1: glucagon-like peptide-1.

c CACS: childhood or adolescent cancer survivor.

d PA: physical activity.

Ten [17-21,25,26,28-30] of the 14 studies included a parent, caregiver, or family component in the target population or intervention design, such as parent-child dyads, family-based obesity management, or studies collecting both child and caregiver perspectives. The remaining 4 [22-24,27] were primarily youth-focused, although family influence was acknowledged as an important contextual factor. The degree of family involvement varied considerably, ranging from minimal parental support or consent to parent-mediated delivery, co-participation in activities, shared monitoring, and parent-facing intervention interfaces.

Reporting of demographic and contextual characteristics was inconsistent. Child age was available in all 14 studies. While parents’ age was reported in only 4 studies [17,19-21]. Female participation or sex distribution was reported in 11 studies [17-24,26,27,29]. Among studies reporting child sex distribution, the proportion of female participants ranged from 16.7% to 65.0% [20,24]. Language or linguistic context could be identified in all 14 studies. However, cultural tailoring was reported in only one study [19], and 4 additional studies [20,21,23,30] described some form of contextual, linguistic, developmental, or population-specific tailoring.

### Intervention Design and Delivery Characteristics

Intervention design and delivery characteristics were presented in Table 3. Mobile app– or smartphone-based delivery was the most common format, appearing in 9 studies [17-19,22,24-27,29]. Six studies [19,20,23,28-30] incorporated a clearly web-based component, such as a dashboard, web platform, online interface, or browser-based system. Other delivery modes included chatbot-supported platforms, WhatsApp (Meta)-supported programming, SMS text messaging or responsive text messaging, serious games, metaverse-supported systems, and hybrid platforms integrating child-, parent-, and clinician-facing tools.

**Table 3.** Intervention design and delivery characteristics.

Study and year		Intervention type	Platform or delivery mode	Intervention duration	Wearable or sensor used	Family involvement	Comparator or Control	Intervention components or behavior change features
Aydemir (2025) [17]		ChatGPT (OpenAI)-delivered, parent-mediated home PA^a^ intervention	ChatGPT+parent training+WhatsApp (Meta) support	4 weeks; 40 min/session; 3 days/week	None	High; parents implemented activities and could involve siblings or family	Non-ChatGPT control group	Parent training; home PA plans; warm-up, main, or cool-down; co-participation; WhatsApp support; rewards
de Arriba Muñoz et al (2026) [18]		Family digital support program adjunct to semaglutide	Adhera Caring Digital Program mobile app (Adhera Health, Inc.)	10-month active intervention; interim results at 150 days	Fitbit Inspire 3 (Google LLC)	High; caregiver-focused education, behavior support, and monitoring	None	Education; motivational messaging; progress feedback; wearable monitoring; psychometrics
Emerson et al (2026) [30]		Adaptation of WeChat (Tencent Holdings Limited) chatbot for ASD^c^ accessibility	Text or email chatbot concept; interviews or focus groups	Not an intervention trial; exploratory qualitative study	Not central; some participants discussed Fitbit or Apple Watch use	High; family-centered care identified as essential	None	SMART^b^ goals; SDOH^d^ screening; health resources; adaptation recommendations; branching logic
Heerman et al (2024) [19]		Digital obesity prevention added to primary care counseling	Responsive text messages+web dashboard+clinic counseling	24 months	None	High; parents were the direct intervention users	Clinic-only counseling group	Goal setting; self-monitoring; tailored feedback; dashboard; growth charts; health literacy materials
Larizza et al (2023) [22]		mHealth^e^ lifestyle improvement app	Smartphone app+physician or admin dashboard+chatbot	2-week home pilot after training	None	High; parents register child, enter data, monitor trends, support use	None	Goals; education; success stories; monthly questionnaires; diaries; badges; quizzes; avatar; chatbot
Moore et al (2024) [23]		Conversational agent to overcome PA barriers	Web or text-based conversational agent	Prototype evaluation; not long-term intervention	None	Low or minimal	None	Barrier identification; confidence and motivation modules; social dialogue; links or resources; personalization
Oh et al (2022) [24]		AI-based interactive home exercise exergame	Smartphone-based gesture-recognition game versus Nintendo Switch Ring Fit (Nintendo Co., Ltd.)	3 weeks; 30 min/session, 5 days/week	Camera or computer vision gesture recognition	Low or minimal	Nintendo Switch exercise game	Gamified exercise; real-time visual or auditory feedback; alarms; posture feedback
Peuters et al (2024) [25]		Multicomponent healthy lifestyle or mental health app	LifeGoals (The University of Michigan) mobile app+Fitbit+videos+chatbot	12 weeks	Fitbit Charge 2/3 (intervention); Axivity accelerometers for measurement	Low or minimal direct family role	No-intervention control group	Goal setting; action or coping planning; self-monitoring; rewards; gamification; narrative videos; chatbot; information
Stasinaki et al (2021) [26]		Conversational agent obesity management app+ reduced on-site counseling	PathMate2 mobile app+clinic visits	5.5-month intensive phase +6-month maintenance (12 mo total)	None mentioned for intervention	Moderate; family part of counseling context but youth-focused app	Standard multicomponent behavior change intervention	Daily chatbot counseling; step or activity challenges; meal photos; breathing exercises; goal setting; educational handouts
Tan et al (2026) [27]		AI-assisted school-based health behavior chatbot or web app	Web-based app on school laptops	8 weeks	None	Low; parental consent but no active family component	None	SMART goals; self-monitoring; behavior logging; reflections; AI feedback; progress charts; daily reports; education
Zarkogianni et al (2023) [29]		Family-based mHealth + AI+serious game obesity management platform	Serious game+child app+parent app+clinician app+activity tracker	3 months (12 wk)	Fitbit Ace 2	High; parent app, personalized messages to mothers, family-based intervention	Active control and intervention versions of platform in pilot sequence	Self-monitoring; serious games; personalized messages; meal plans; PA and sleep tracking; clinician/nutritionist support
Kim et al (2025) [20]		Smart health care service design using metaverse, chatbot, wearable, and web or app ecosystem	Student app+parent app+teacher web+metaverse+chatbot+wearable	Development study; no intervention exposure period	Planned wearable device or smartwatch integration	High; parent app and parent participation central in service design	None	Tracking; personalized services; gamification; rewards; consultation; school and parent interfaces
Kang et al (2026) [21]		Cross-sectional assessment of healthy lifestyle practices and acceptability of AI chatbot use for future lifestyle management; no active intervention delivered	Online web-based survey (Google Forms); chatbot was discussed conceptually, not deployed	Cross-sectional survey	None	High for assessment purposes; survivor and parent perspectives were both collected to inform future family-tailored chatbot support	Survivors versus parents (comparative groups); no intervention control	Healthy lifestyle assessment across 7 domains; chatbot awareness or acceptability assessment; open-ended unmet-need responses analyzed with topic modeling
Willms and Liu (2024) [28]		JITAI^f^ content development using ChatGPT	ChatGPT-3+ Pathverse no-code mobile app builder	10-week planned JITAI; development process over 2 months	Intended JITAI used child MVPA^g^ minutes as decision input; no specific wearable evaluated in case study	High; parent supports child PA	None	Tailored lessons; decision-tree personalization; family PA challenges

a PA: physical activity.

b SMART: Specific, Measurable, Achievable, Relevant, and Time-bound.

c ASD: autism spectrum disorder..

d SDOH: Social determinants of health

e mHealth: mobile health.

f JITAI: Just-in-Time Adaptive Intervention.

g MVPA: moderate to vigorous physical activity.

Nine studies [17-19,22,24-27,29] reported a defined intervention or exposure period, with durations ranging from 2 weeks to 24 months. Four studies [17,24,25,27] had relatively short intervention periods of 2-4 weeks, 3 [22,26,29] lasted approximately 8-12 weeks, and 2 [18,19] extended beyond 5 months, including one 24-month randomized trial and one 12-month obesity management program. The remaining 5 studies [20,21,23,28,30] were qualitative, developmental, survey-based, or case-based and therefore did not involve a conventional intervention period.

Most interventions were multicomponent and incorporated established behavior change strategies. Self-monitoring or tracking components were present in 9 studies [18-22,25-27,29], personalized feedback or adaptive content in 10 studies [17-20,23,25-27,29,30], and education or informational support in 9 studies [18,19,21-23,25-27,30]. Goal setting or challenge-based components were identified in 7 studies [19,22,25-28,30], gamification or reward-related elements in 7 studies [17,20,22,24-26,29], and social support, co-participation, or human-supported features in 8 studies [17-20,23,25,29,30]. Reminders or prompts were described as a distinct feature in 4 studies [17,24,26,27]. Although all studies were relevant to PA, only 5 [17,23,24,27,28] focused primarily on PA or exercise promotion; the remaining 9 [18-22,25,26,29,30] embedded PA within broader obesity-management, lifestyle, rehabilitation, or healthy living interventions.

Wearable devices, sensor-based inputs, or objective digital monitoring were reported in 6 studies [18,24,25,27-29]. These included Fitbit (Google LLC) devices, accelerometers, computer vision–based gesture recognition, smartphone- or app-linked monitoring, and planned smartwatch integration. However, most studies still relied partly or primarily on self-reported behavior, questionnaires, or user-entered information.

### AI Features and Functional Characteristics

The included studies varied substantially in the sophistication and reporting of their AI and automation features (Table 4). To improve clarity, we distinguished the named intervention system or platform from the reported AI technique or automation logic. Three studies [17,27,28] used generative AI or large language model–based approaches directly in intervention delivery or content development. Several studies used scripted, predefined, or automated chatbots and tailoring functions based on structured pathways, branching logic, or automated rules [19,22,25,26,30]. Moore et al [23] described natural language understanding for intent recognition and dialogue routing. The study by de Arriba Muñoz et al [18] used a hybrid personalization engine, Zarkogianni et al [29] used a machine learning–based recommendation system, Oh et al [24] used computer vision and deep learning for gesture recognition, and Kim et al [20] described a broader integrated chatbot-supported digital health ecosystem.

**Table 4.** AI features and functional characteristics of the included interventions.

Study	Year	AI or automation role	Intervention system or platform	Reported AI technique or automation logic	Input data type	AI task	AI contribution/claimed benefit	AI explainability/transparency reported
Aydemir [17]	2025	Generates individualized home physical activity plans and answers parent follow-up questions	ChatGPT-4 (OpenAI)	Generative AI using a large language model	Parent text prompts; child age; and ASD^b^ status	Text generation; PA^a^ recommendation; instructional clarification	Low-cost, home-based, scalable PA guidance for ASD families	Limited; prompts described but model logic not detailed
de Arriba Muñoz et al [18]	2026	Personalizes caregiver support content	Adhera AI Precision Digital Companion (Adhera Health, Inc)	Hybrid personalization engine; specific algorithm not fully reported	App engagement data; psychometrics; wearable metrics; clinical data	Personalization; feedback; progress support	Complement pharmacotherapy with scalable caregiver support and monitoring	Limited
Emerson et al [30]	2026	Delivers health prompts, screening questions, and resources	WE CHAT (Wellness Education to Create Healthy Habits and Actions to Thrive)	Predefined chatbot pathways or branching logic; underlying algorithm not fully reported	User responses to prompts; provider recommendations for adaptation	Goal support; screening; resource/referral prompts	Potential to support obesity prevention for youth with ASD if adapted for variability and caregiver burden	Moderate; logic and use described conceptually
Heerman et al [19]	2024	Provides automated tailoring and adaptive feedback	Greenlight Plus digital platform (Vanderbilt University Medical Center)	Automated tailoring using predefined logic; no machine learning model or large language model reported	Parent surveys; goal progress self-ratings; child anthropometrics	Tailored messaging; adaptive feedback; dashboard tracking	Frequent asynchronous support and scalable tailored obesity prevention	Moderate; tailored logic described
Larizza et al [22]	2023	Collects responses and provides practical advice through a virtual coach	V-care app (University of Pavia) with Google Dialogflow chatbot	Task-oriented conversational agent using Dialogflow natural language processing and predefined workflows	Profile, questionnaires, quizzes, daily/weekly behavior diary, BMI updates	Questionnaire administration; coaching; advice; engagement	Increase compliance and reduce dropout in pediatric obesity management	Good technical description of architecture; limited algorithmic detail beyond workflow
Moore et al [23]	2024	Identifies physical activity barriers and delivers tailored relational support	Phyllis conversational agent (Sheffield Hallam University); Google Dialogflow and MindBehind (MindBehind Inc) engine	Natural language understanding for intent recognition and dialogue routing	Open-text user barrier input; conversation responses	Intent recognition; routing; tailored dialogue; persuasive coaching	On-demand personalized support to overcome PA barriers	Moderate; NLU^c^ training and *F*_1_-score reported
Oh et al [24]	2022	Recognizes body movements and provides exergame feedback	Super Kids Adventure (Funrehab)	Computer vision and deep learning using a convolutional neural network (CNN)	Video/image-based body movement data	Gesture recognition; exercise scoring; real-time feedback	Low-cost motivational home exercise alternative	Basic model type reported (CNN), limited technical detail
Peuters et al [25]	2024	Delivers automated chatbot support and behavior-change content	#LIFEGOALS mobile app chatbot	Scripted or predefined chatbot support; underlying algorithm not fully reported	Self-report survey data; Fitbit (Google LLC)/self-monitoring data; accelerometer data	Support messages; information delivery; self-regulation support	Engage adolescents in healthy lifestyle behavior change and mental health promotion	Limited
Stasinaki et al [26]	2021	Delivers daily conversational coaching and challenges	PathMate2 (Pathmate Technologies AG) mobile app	Scripted or predefined conversational coaching and challenge-delivery logic; underlying algorithm not fully reported	Chat interactions; challenge completion; self-reported meal photos and exercises	Conversational coaching; reminders; challenge monitoring	Low-threshold obesity support with fewer clinic visits	Moderate; app functions described
Tan et al [27]	2026	Provides personalized feedback for self-management	ProudMe Tech (ProudMe Technologies); GPT-4 (OpenAI) API	GPT-assisted chatbot using generative AI	Self-reported goals, behavior logs, reflections, interaction logs	Feedback generation; supportive prompting; text analysis for evaluation	Scalable, low-cost personalized behavioral counseling for students	Partial; app functions described but limited model detail
Zarkogianni et al [29]	2023	Generates personalized messages and recommendations based on user profiles	ENDORSE (ENDORSE Consortium) platform	Machine learning–based recommendation and personalization system, including genetic algorithm components	Tracker data; serious game interactions; clinical/nutrition data; user profile	Personalization; recommendation; risk/behavior profiling	Coordinated family-clinician ecosystem for personalized obesity management	Moderate; AI-based model described at high level
Kim et al [20]	2025	Provides chatbot-supported health guidance and personalized service delivery	MUZZIM smart health service concept	Integrated chatbot-supported digital health ecosystem; specific technical approach not fully reported	Health information, wearable/lifelog data, user needs, app inputs	Guidance; personalization; integrated monitoring	Support sustainable adolescent health habits through school-family digital ecosystem	Conceptual only
Kang et al [21]	2026	Examines the acceptability of a proposed chatbot for future lifestyle management support	No chatbot was deployed or named	Proposed generative AI chatbot concept; no live AI system evaluated	Survey responses on healthy lifestyle practices, chatbot awareness, and open-ended unmet healthy lifestyle needs	Conceptual health education, lifestyle guidance, interaction, and support for future survivorship care	Potential to provide tailored, interactive, real-time support for holistic survivorship healthy lifestyle management	Not applicable because no live AI system was tested
Willms and Liu [28]	2024	Generates intervention content for a just-in-time adaptive intervention	ChatGPT-3 and Pathverse (Pathverse Ltd) no-code mobile app builder	Generative AI using a large language model for content development; predefined decision rules for intervention tailoring	Researcher prompts with theory, target behavior, and intervention structure	Content generation	Rapid, scalable creation of tailored mHealth^d^/JITAI^e^ content	Prompting process described; acknowledged hallucination/privacy limits

a PA: physical activity.

b ASD: autism spectrum disorder.

c NLU: natural language understanding.

d mHealth: mobile health.

e JITAI: Just-in-Time Adaptive Intervention.

Across studies, the most common role of AI was to support personalization and automated interaction rather than diagnosis or prediction. Tailored feedback, adaptive support, or personalized recommendations were described in 10 studies [17-20,22,23,25-27,29]. Chatbot-based or conversational coaching features were present in 8 studies [17,21-23,25-27,29], activity generation or exercise recommendation in 4 studies [17,24,28,29], and monitoring or self-management support in 6 studies [18,19,22,25,26,29].

Input data were most commonly participant- or caregiver-entered, including text prompts, questionnaires, goal selections, behavior logs, profile information, or interaction history. Six studies [18,24,25,27-29] incorporated wearable, sensor, image, or other objectively tracked data as part of the AI-supported system. Technical reporting of AI methods was often limited. Seven studies [20,21,23,27-30] provided only limited, conceptual, or partial descriptions of AI logic or architecture, whereas 6 [17-19,22,24,25] provided at least moderate detail about system workflow, personalization logic, or model type. One study [21] did not evaluate a live AI system but instead assessed attitudes toward a proposed future chatbot.

### Theory, Implementation, and Outcome Findings

Theoretical frameworks were identified in most studies (Table 5), including Social Cognitive Theory [19]; the capability, opportunity, motivation, and behavior (COM-B) model; the Theoretical Domains Framework [23]; the Health Action Process Approach; the Elaboration Likelihood Model; Persuasive Systems Design [25]; Self-Determination Theory [27,29]; the Health Promotion Model; the Technology Acceptance Model [21], and the Multi-Process Action Control (M-PAC) model [28]. Other studies referenced broader approaches such as cognitive-behavioral principles, family-centered care, or user-centered design, whereas Aydemir [17] and Oh et al [24] did not report an explicit theoretical framework.

**Table 5.** Theoretical foundations, implementation outcomes, and behavioral/health outcomes.

Study and year		Theoretical framework	Feasibility/acceptability/usability/engagement	Primary PA^a^ outcomes	Secondary outcomes	Key findings/effect Size
Aydemir (2025) [17]		NR^b^	High parent satisfaction; 92% participated in WhatsApp (Meta) discussions	Leisure Time Exercise Questionnaire (LTEQ)	Parent feasibility feedback	LTEQ increased from 6.69 to 34.00 in intervention versus 7.46-7.23 in control; interaction *F*=353.07; *P*<.001; ηp²=.936
de Arriba Muñoz et al (2026) [18]		Cognitive-behavioral principles	SUS^c^ score 72; exploratory trends between higher engagement and better fat loss	Wearable-recorded PA/biometric metrics collected, but main reported outcomes were clinical obesity measures	BMI, weight, body fat %, muscle mass %, waist circumference, usability, and engagement	At Day 150: BMI –4.51 kg/m²; weight –11.42 kg; body fat –5.63%; waist –8.69 cm; muscle mass 4.47% (all *P*<.001)
Emerson et al (2026) [30]		Family-centered care- or user-centered adaptation	Qualitative evidence of interest and need; branching logic and family support emphasized	NR	Feasibility, acceptability, accessibility needs, and adaptation recommendations	No efficacy outcomes; key themes were ASD^d^ variability, perceived value of mHealth^e^, family-centered care, and interdisciplinary support
Heerman et al (2024) [19]		Social Cognitive Theory; health literacy-informed design	High retention: 86.3% primary outcome at 24 months	No direct PA primary outcome; PA support behaviors embedded in program	Weight-for-length trajectory; BMI *z* score; overweight/obesity prevalence	Weight-for-length at 24 mo was lower by –0.33 kg/m (95% CI –0.57 to –0.09); BMI *z* score −0.19; obesity 7.4% versus 12.7% (aRR^f^ 0.56)
Larizza et al (2023) [22]		Psychological/behavioral change theories; goal-based behavioral intervention	Overall friendliness 85%; perceived utility 100%; chatbot acceptable to 69%; all average scores >3/5	No quantitative PA outcome reported in pilot	Usability, acceptability, friendliness, and utility	High usability and perceived utility; chatbot section scored lower than other features but remained >3/5
Moore et al (2024) [23]		COM-B^g^; Theoretical domains framework; behavior change techniques; theory of planned behavior for confidence module	61 conversations; positive UX^h^; 73% definite intention to use; *F*_1_-score about 80%	Perceived confidence and motivation to engage in PA	Acceptability, usability, recommendation likelihood, and barrier coverage	Significant increase in self-reported confidence and motivation after interacting with Phyllis (Sheffield Hallam University); 73% would use future version
Oh et al (2022) [24]		NR	Postquestionnaire on motivation, fun, perceived exercise effectiveness	Calorie expenditure; VO_2_max^i^; 6MWT^j^; RPE^k^; BMI	Motivation, fun, and perceived exercise effectiveness	SUKIA (Funrehab) showed superior calorie consumption, VO*_2_*max, and RPE versus Nintendo Switch (Nintendo Co, Ltd); authors report better cardiopulmonary function and energy expenditure
Peuters et al (2024) [25]		Health action process approach; elaboration likelihood model; persuasive systems design	High nonusage attrition: 18% never used app; 30% stopped by week 2; interviews highlighted rewards and self-regulation as helpful	PA (ENMO^l^/MVPA^m^) and sedentary behavior from accelerometers	HRQoL^n^, mood, peer support, sleep quality, sleep routine, and breakfast	Beneficial effects for PA (*χ*²=4.36; *P*=.04), sedentary behavior (*χ*²=6.44; *P*=.01), sleep quality (*χ*²=6.11; *P*=.01), and mood (*P*=.02); effects moderated by COVID restrictions
Stasinaki et al (2021) [26]		Behavior change techniques; obesity counseling framework (no single explicit theory emphasized)	Average daily app usage 71.5%; no side effects observed	Physical capacities (strength, agility, and endurance) via modified Dordel-Koch test	BMI-SDS^o^, fat mass, muscle mass, waist-to-height ratio, BP^p^, pulse, and stress markers	BMI-SDS decreased significantly in controls at 5.5 months, not in PathMate2 (PathMate Technologies GmbH); both groups improved muscle mass, strength, and agility by 12 months; PathMate2 reduced fat mass at T1/T2
Tan et al (2026) [27]		Self-Determination Theory; Self-Regulation Theory	Mean 8.9 behavior entries, 30 reflections, 33.5 AI feedback messages; 63.8% of daily goals achieved	Self-reported PA hours	Screen time, fruit/vegetable intake, sleep, engagement, and sentiment	PA did not significantly change (2.5-2.4 h/day; *P*=.20); screen time decreased by 0.93 h/day (*P*<.001); fruit/veg intake decreased by 0.50 servings (*P*=.02); sleep unchanged
Zarkogianni et al (2023) [29]		Self-Determination Theory	Adherence tracked by usage frequency; activity tracker use correlated with BMI improvement	Activity tracker usage / PA patterns (minutes of activity; steps/day)	BMI *z* score, diet quality, screen time, sleep duration, and usability/adherence	Mean BMI *z* score reduction –0.21 (SD 0.26); *P*<.001; tracker usage correlated with BMI *z* score improvement (*r*=−0.355*; P*=.02)
Kim et al (2025) [20]		MASUN^q^ framework/user-centered design methodology	Students/parents prioritized PA and sleep tracking; reward systems rated promising; teachers rated effectiveness/usefulness/usability highly	NR	User needs, usability ratings, and preferred features	No efficacy outcomes; stakeholders favored PA measurement, rewards, and integrated monitoring across student-parent-teacher interfaces
Kang et al (2026) [21]		Health Promotion Model and Technology Acceptance Model	Positive perceptions of chatbot use in both groups; mean A-uC^r^ scores were above 4/5 overall, with parents showing higher intention to use	No intervention PA outcome; PA was one healthy lifestyle subdomain and a commonly reported unmet need	Overall healthy lifestyle score and subdomains; awareness of chatbot use (perceived usefulness/ease, value, empathy, reliability, professionalism, intention to use); topic-modeled unmet needs	No significant group differences in overall healthy lifestyle score (CACSs^s^ mean 3.16, SD 0.80 vs parents mean 3.18, SD 0.36; *P*=.74) or PA subscale (mean 2.55, SD 0.89 vs mean 2.53, SD 0.81, *P*=.91). Parents had higher intention to use chatbots (mean 4.21, SD 0.68 vs mean 3.94; SD 0.90; *P*=.03). Common unmet areas were exercise, healthy diet, and regular lifestyle.
Willms and Liu (2024) [28]		Multi-Process Action Control (M-PAC)	Researchers found ChatGPT acceptable and easy to use for producing 13 lessons	NR	Feasibility, acceptability, and ease of use of AI-generated content process	ChatGPT (OpenAI) was judged acceptable and easy to use for creating 13 PA JITAI^t^ lessons, but expert review remained essential due to the risk of inaccurate references/content

a PA: physical activity.

b NR: Not Reported

c SUS: system usability scale.

d ASD: autism spectrum disorder.

e mHealth: mobile health.

f aRR: adjusted risk ratio.

g COM-B: capability, opportunity, motivation, and behavior.

h UX: user experience.

i VO₂max: maximal oxygen uptake.

j 6MWT: 6-minute walk test.

k RPE: rating of perceived exertion.

l ENMO: Euclidean norm minus one.

m MVPA: moderate to vigorous physical activity.

n HRQoL: Health-related quality of life.

o BMI-SDS: body mass index standard deviation score.

p BP: blood pressure.

q MASUN: Method of App Selection based on User Needs.

r A-uC: Awareness of the Use of Chatbots.

s CACS: childhood or adolescent cancer survivor.

t JITAI: Just-in-Time Adaptive Intervention.

Outcome assessment approaches varied across studies (Table 5). Six studies [18,19,24,25,27,29] included an objective or performance-based PA-related measure, such as accelerometer-assessed activity, Fitbit-derived metrics, step counts, calorie expenditure, physical fitness testing, or device-recorded movement patterns. Four studies [17,21,23,26] relied primarily on self-report or parent-report PA-related measures, including exercise questionnaires, activity hours, or perceived confidence and motivation for PA. The remaining 4 studies [20,22,28,30] did not directly assess intervention-related PA outcomes.

The studies frequently examined outcomes beyond PA alone. Anthropometric or weight-related outcomes, including BMI, BMI *z* score, body fat, waist circumference, or obesity-related indicators, were reported in 6 studies [18,19,22,24,26,29]. Diet- or nutrition-related outcomes were reported in 4 studies [18,19,21,29], screen time or sedentary behavior in 3 studies [19,25,27], and sleep-related outcomes in 3 studies [19,21,25]. Psychosocial, motivational, quality-of-life, and usability-related outcomes were also commonly reported, suggesting that many AI-supported family-based interventions were conceptualized as broader healthy lifestyle or obesity-management programs rather than narrowly focused PA interventions.

Among the 8 studies that reported direct PA-, exercise-, movement-, or fitness-related outcomes, 4 [17,23-25] reported significant improvements in PA participation, exercise performance, movement-related behavior, or fitness-related indicators. Two studies [26,27] reported little or no statistically significant improvement in PA despite favorable findings for engagement or other health outcomes. One study [18] collected wearable-based PA data but reported stronger clinical obesity-related effects than PA-specific effects, and one additional study [19] described PA-related findings as part of a broader multibehavior framework without demonstrating a clearly significant PA effect.

All 14 original studies reported at least one feasibility-, acceptability-, usability-, or engagement-related outcome (Table 5). Five studies [20,21,23,28,30] were primarily qualitative, developmental, survey-based, or case-based in design and therefore emphasized user preferences, accessibility needs, perceived usefulness, desired features, implementation barriers, or design priorities rather than behavioral efficacy. These studies generally reported favorable perceptions of AI-supported tools, particularly when interventions were viewed as personalized, interactive, and supportive of family routines or youth autonomy.

Among the intervention-oriented studies, quantitative implementation indicators were commonly reported. Examples included high parent satisfaction and 92% participation in WhatsApp discussions in one parent-mediated ChatGPT study [17]; a System Usability Scale score of 72 in one obesity-management intervention [18]; 86.3% retention at 24 months in the large primary care trial; 85% friendliness, 100% perceived utility, and 69% acceptability in one pediatric obesity app pilot [22]; 61 total chatbot conversations and 73% definite intention to use in one conversational-agent co-design study [23]; average daily app usage of 71.5% in one obesity management trial [26]; and a mean of 8.9 (SD 7.6) behavior entries, 30 reflections, and 33.5 AI feedback interactions in one school-based chatbot study [27]. One quasi-randomized trial reported substantial nonuse attrition, with 18% of participants never using the app and 30% stopping use by week 2 [25].

## Discussion

### Principal Findings

This scoping review explored the emerging evidence on AI and chatbot-supported interventions targeting PA and obesity-related lifestyle behaviors among children and adolescents, with attention to family involvement. Across the 14 studies included, the evidence indicates that AI-supported interventions are being used in increasingly diverse ways to support PA, obesity management, and related health behaviors in pediatric populations, but the literature remains varied in terms of intervention design, AI capabilities, family involvement, and outcome measurement.

### Complex AI Features Across Studies

Across the included studies, AI features span a continuum of complexity, from rule-based scripted chatbots and automated tailoring systems (eg, structured coaching or tailored text messaging) to hybrid systems that incorporated limited natural language understanding or personalization. AI was most commonly used to support personalization, user interaction, and ongoing behavioral support, rather than for diagnostic, predictive, or decision-making purposes. Many studies provided only high-level or conceptual descriptions of system functionality, with limited detail on model architecture, training data, validation procedures, or performance across subgroups. This lack of transparency makes it difficult to assess reproducibility, evaluate potential bias, or determine whether AI-driven personalization meaningfully contributes to outcomes beyond standard digital tailoring approaches. These gaps are particularly important given the increasing integration of more complex AI systems, which introduce additional concerns related to accuracy, safety, and algorithmic bias. Future research should adopt AI-specific reporting standards and explicitly document how AI outputs are validated, monitored, and updated over time across diverse populations.

### Delivery Platforms and Intervention Components

Regarding the delivery platform and intervention components, most interventions embed common behavior change functions, including self-monitoring, goal setting, education, feedback, and rewards, within various platforms. These platforms range from low-burden text messaging and web dashboards to multiapp family–clinician ecosystems, wearable-linked systems, higher-complexity immersive environments, and sensor-rich exercise apps. Engagement strategies also include gamification, relational dialogue, narrative content, and school- or family-embedded routines. Within this structure, AI primarily serves as a delivery and personalization mechanism that shaped how these components were implemented. AI enabled more dynamic forms of support, such as routine coaches, on-demand assistants, and interactive educational or motivational agents [23,25,28]. In this sense, AI modifies the timing, intensity, and responsiveness of intervention delivery rather than fundamentally changing its core behavioral content.

### Extent and Operationalization of Family Engagement

A central contribution to this review is the characterization of family involvement, which was important but inconsistently operationalized. In some studies, parents were critical to implementation, such as delivering activities at home, receiving caregiver education, or interacting with the digital system directly [17-19,29]. In others, youth were the primary users and family influence was acknowledged but not fully integrated into the intervention structure [23,24,27]. This variability suggests that the field has not yet converged on a clear definition of what constitutes a “family-based” AI intervention. Importantly, most studies do not measure family engagement systematically enough to test whether caregiver involvement mediates or moderates intervention effects. More precise conceptualization of whether caregivers are intended as co-users, facilitators, role models, or targets of behavior change may improve both intervention design and interpretation of outcomes.

### Limited Cultural Tailoring and Bilingual Intervention

With respect to cultural tailoring and bilingual delivery, evidence remains limited. One large randomized clinical trial embedded bilingual delivery (English/Spanish) and cultural appropriateness processes in a racially and ethnically diverse population [19]. Although all studies could be situated within identifiable linguistic or contextual settings, specific cultural adaptation was rare, and the evidence base was entirely in middle- and high-income countries. This limits the transferability of current findings to lower-resource contexts and to culturally diverse family populations whose routines, values, digital access, and barriers to health behavior change may differ substantially. The lack of consistent reporting on cultural tailoring, bilingual adaptation, and structural access issues also limits understanding of how well current interventions align with the realities of underserved families. These limitations introduce broader concerns in digital health that technologies may inadvertently reinforce inequities if they are developed primarily in well-resourced settings without sufficient adaptation for different social and cultural contexts [31].

### Exploratory Feasibility and Efficacy Findings

Consistent with the aims of this review, PA emerged as the most prominent target behavior, either as a primary intervention focus or as part of broader multibehavior lifestyle and obesity-management programs. Several studies focused directly on PA promotion, home exercise, or movement-related confidence and performance [17,23,24,27], whereas others addressed PA within broader interventions targeting obesity prevention, rehabilitation, or healthy lifestyle routines [18,19,25,26,29]. This pattern likely reflects both the importance of PA in pediatric health promotion and the broader reality that children’s movement behaviors are closely associated with diet, sleep, sedentary behavior, and family routines. Rather than treating PA as an isolated outcome, many of the included studies positioned it within a more comprehensive family lifestyle framework.

The reviewed studies also indicate that AI-supported interventions were generally more consistent in demonstrating feasibility, acceptability, and engagement than in behavioral efficacy. These findings align with previous youth-focused syntheses emphasizing that chatbots and AI-supported tools are often perceived as appealing and acceptable when they are interactive, personalized, and low burden [12]. However, the current review also found that favorable usability does not necessarily translate into sustained engagement or robust changes in PA participation, exercise performance, calorie expenditure, cardiopulmonary function, or confidence and motivation for PA [17,23-25], and some broader obesity-management or prevention interventions also showed favorable changes in clinical or anthropometric outcomes [18,19,29]. At the same time, findings were not uniform across studies. Tan et al [27] did not detect significant changes in PA, and Stasinaki et al [26] did not show clear superiority of the conversational-agent intervention over the comparison condition for BMI-SD score (BMI-SDS). Moreover, many included studies were feasibility, pilot, or developmental studies rather than adequately powered effectiveness trials. These findings suggest that while AI-supported interventions show promise, the current evidence remains stronger for feasibility and acceptability than for clinically meaningful and sustained improvements in PA and related outcomes.

### Implications for Practice and Research

This review highlights several implications for intervention design, implementation, and future research. In practice, AI-supported family interventions may be most useful when integrated into broader family-based, school-based, or clinical programs rather than positioned as isolated tools. Their strengths appear to lie in delivering tailored education, self-monitoring support, reminders, coaching, and feedback in ways that are flexible, scalable, and responsive to daily family routines. However, the findings also indicate that intervention success likely depends on more than the presence of AI alone. Additional attention to developmental appropriateness, caregiver roles, response quality, burden of use, and contextual relevance will be essential to sustaining engagement and producing meaningful behavior change.

Future research and program development should prioritize family-centered co-design, more rigorous comparative and longitudinal study designs, and greater consistency and transparency in reporting AI features, personalization logic, and behavior change techniques. More culturally and linguistically responsive adaptation is also needed, particularly for diverse and underserved family populations. Additional work should examine implementation outcomes such as reach, adoption, maintenance, scalability, and cost, alongside behavioral and clinical outcomes. Emerging approaches such as generative AI, wearable-integrated personalization, and adaptive feedback systems may offer added value, but their safety, accuracy, transparency, and practical benefit require more careful evaluation in pediatric settings. Continued interdisciplinary collaboration will play an important role in ensuring that AI-supported family interventions are not only innovative but also ethical, accessible, and equitable.

### Limitations

Several limitations should be acknowledged. First, the number of included studies was small, and the evidence base was highly heterogeneous. Further, the included studies varied widely in population, intervention modality, duration, AI sophistication, and measured outcomes, which limits comparability. In addition, many studies were pilots, feasibility studies, or usability-focused investigations rather than adequately powered trials. Further, reporting of demographic, contextual, and technical AI details was often incomplete, making it difficult to assess generalizability or identify active intervention ingredients. The search was limited to English-language publications; therefore, relevant studies published in other languages may not have been captured, which may have introduced selection bias. Further, the search was limited to peer-reviewed publications indexed in the selected databases and did not include a formal gray-literature search or supplementary citation tracking. Therefore, relevant evidence reported in theses, reports, conference materials, policy documents, unpublished sources, or publications not indexed in the selected databases may not have been captured. A formal critical appraisal or methodological quality assessment was not conducted; therefore, the methodological rigor of the included studies was not evaluated.

### Conclusion

In conclusion, this scoping review found that AI and chatbot-supported interventions for pediatric PA and obesity-related lifestyle behaviors are a rapidly emerging but still early-stage field. The existing literature suggests that these interventions are generally feasible, acceptable, and promising as scalable tools for supporting healthy behavior change among children and families. However, evidence for effectiveness, particularly for PA outcomes, remains mixed and limited by methodological heterogeneity, short follow-up, and inconsistent outcomes. The review also highlights important gaps in family-based design, cultural tailoring, equity, and evaluation of advanced AI capabilities. Future research should prioritize rigorous trials, clearer conceptualization of family engagement, objective outcome measurement, and culturally responsive development to ensure that AI-supported interventions are not only innovative but also effective, equitable, and meaningful for diverse families.

## Supplementary material

10.2196/98889 Multimedia Appendix 1 Search terms by databases.

10.2196/98889 Checklist 1 PRISMA-ScR checklist.

## Abbreviations

BMI-SDS: BMI-SD score
COM-B: capability, opportunity, motivation, and behavior
M-PAC: Multi-Process Action Control
PA: physical activity
PICOS: population, intervention, comparator, outcomes, and study design
PRISMA: Preferred Reporting Items for Systematic Reviews and Meta-Analyses
PRISMA-ScR: Preferred Reporting Items for Systematic Reviews and Meta-Analyses extension for Scoping Reviews
WHO: World Health Organization
