# Supplementary material for: AI and Chatbot-Supported Interventions for Physical Activity and Obesity-Related Lifestyle Behaviors: Scoping Review With Attention to Family Involvement
Source: JMIR Pediatr Parent. 2026 Jul 24;9:e98889. doi: 10.2196/98889 (PMC13399406; doi:10.2196/98889)
Supplement: Multimedia Appendix 1 [file pediatrics-v9-e98889-s001.docx]

Appendix 1. Search terms by database

| Database | Search term | Search date | Results | Filter | Original |
| --- | --- | --- | --- | --- | --- |
| pubmed | ( chatbot*[tiab] OR "chat bot*"[tiab] OR "conversational agent*"[tiab] OR "virtual agent*"[tiab] OR "relational agent*"[tiab] OR "virtual assistant*"[tiab] OR "digital assistant*"[tiab] OR "AI chatbot*"[tiab] OR "AI coach*"[tiab] OR "virtual coach*"[tiab] OR "conversational AI"[tiab] OR chatgpt[tiab] OR "large language model*"[tiab] OR LLM*[tiab] OR "generative AI"[tiab] ) AND ( "physical activ*"[tiab] OR exercise*[tiab] OR "active living"[tiab] OR obes*[tiab] OR obesity[tiab] OR overweight[tiab] OR "weight management"[tiab] OR "obesity prevention"[tiab] OR "screen"[tiab] OR "sedentary"[tiab]) AND ( child*[tiab] OR adolescen*[tiab] OR youth[tiab] OR teen*[tiab] OR pediatric*[tiab] OR paediatric*[tiab] OR famil*[tiab] OR parent*[tiab] OR caregiver*[tiab] OR mother*[tiab] OR father*[tiab] OR "family-based"[tiab] OR "parent-based"[tiab] ) NOT (review[pt] OR meta-analysis[pt]) | 2/8/26 | 87 | last 10 years, not review, meta-analysis, systematic review | 116 |
| web of science | (chatbot* OR "chat bot*" OR "conversational agent*" OR "virtual agent*" OR "relational agent*" OR "virtual assistant*" OR "digital assistant*" OR "AI chatbot*" OR "AI coach*" OR "virtual coach*" OR "conversational AI" OR chatgpt OR "large language model*" OR LLM* OR "generative AI") AND ("physical activ*" OR exercise* OR "active living" OR obes* OR obesity OR overweight OR "weight management" OR "obesity prevention") AND (child* OR adolescen* OR youth OR teen* OR pediatric* OR paediatric* OR famil* OR parent* OR caregiver* OR mother* OR father* OR "family-based" OR "parent-based") | 2/8/26 | 168 | last 10 years, not review article | 220 |
| psycinfo | ( chatbot* OR "chat bot*" OR "conversational agent*" OR "virtual agent*" OR "virtual assistant*" OR "relational agent*" OR "AI chatbot*" OR "AI coach*" OR "virtual coach*" OR chatgpt OR "generative AI" OR "large language model*" OR LLM* ) AND ( "physical activ*" OR exercise* OR obes* OR obesity OR overweight OR "weight management" OR "obesity prevention" OR "screen" OR "sedentary" ) AND ( child* OR adolescen* OR youth OR teen* OR pediatric* OR paediatric* OR famil* OR parent* OR caregiver* ) | 2/8/26 | 46 | last 10 years | 46 |
| Academic search complete | ( chatbot* OR "chat bot*" OR "conversational agent*" OR "virtual agent*" OR "virtual assistant*" OR "relational agent*" OR "AI chatbot*" OR "AI coach*" OR "virtual coach*" OR chatgpt OR "generative AI" OR "large language model*" OR LLM* ) AND ( "physical activ*" OR exercise* OR obes* OR obesity OR overweight OR "weight management" OR "obesity prevention" OR "screen" OR "sedentary") AND ( child* OR adolescen* OR youth OR teen* OR pediatric* OR paediatric* OR famil* OR parent* OR caregiver* ) | 2/8/26 | 105 | last 10 years | 105 |
| CINAHL Ultimate | ( chatbot* OR "chat bot*" OR "conversational agent*" OR "virtual agent*" OR "virtual assistant*" OR "relational agent*" OR "AI chatbot*" OR "AI coach*" OR "virtual coach*" OR chatgpt OR "generative AI" OR "large language model*" OR LLM* ) AND ( "physical activ*" OR exercise* OR obes* OR obesity OR overweight OR "weight management" OR "obesity prevention" OR "screen" OR "sedentary" ) AND ( child* OR adolescen* OR youth OR teen* OR pediatric* OR paediatric* OR famil* OR parent* OR caregiver* ) | 2/8/26 | 41 | last 10 years | 41 |
| IEEE Xplore | ("chatbot" OR "chat bot" OR "conversational agent" OR "virtual coach" OR "virtual assistant" OR "AI coach" OR "ChatGPT" OR "generative AI") AND ("physical activity" OR exercise OR obesity OR overweight OR "weight management") AND (child OR adolescent OR youth OR pediatric OR family OR parent OR caregiver) | 2/8/26 | 32 | last 10 years | 32 |
| Scopus | ( chatbot* OR "chat bot*" OR "conversational agent*" OR "virtual agent*" OR "relational agent*" OR "virtual assistant*" OR "digital assistant*" OR "AI chatbot*" OR "AI coach*" OR "virtual coach*" OR "conversational AI" OR chatgpt OR "large language model*" OR LLM* OR "generative AI" OR "AI" OR "mHealth" ) AND ( "physical activ*" OR exercise* OR "active living" OR obes* OR obesity OR overweight OR "weight management" OR "obesity prevention" OR "screen" OR "sedentary" OR "active travel" OR "travel mode" OR "walk" OR "trave" ) AND ( child* OR adolescen* OR youth OR teen* OR pediatric* OR paediatric* OR famil* OR parent* OR caregiver* OR mother* OR father* OR "family-based" OR "parent-based" OR "provider" ) | 2/8/26 | 2251 | last 10 years | 2975 |
